# Carotid plaque and bone density and microarchitecture in psoriatic arthritis: the correlation with soluble ST2

**DOI:** 10.1038/srep32116

**Published:** 2016-08-24

**Authors:** Jiayun Shen, Qing Shang, Chun-Kwok Wong, Edmund K. Li, Emily W. Kun, Isaac T. Cheng, Martin Li, Tena K. Li, Tracy Y. Zhu, Cheuk-Man Yu, Ling Qin, Lai-Shan Tam

**Affiliations:** 1 Department of Medicine & Therapeutics, The Prince of Wales Hospital, The Chinese University of Hong Kong, Hong Kong; 2 Department of Chemical Pathology, The Prince of Wales Hospital, The Chinese University of Hong Kong, Hong Kong; 3 Department of Medicine and Geriatrics, Taipo Hospital, Hong Kong; 4 Bone Quality and Health Center of the Department of Orthopedics & Traumatology, The Prince of Wales Hospital, The Chinese University of Hong Kong, Hong Kong

## Abstract

Psoriatic arthritis (PsA) patients have increased risk of both atherosclerosis and osteoporosis. Previous studies revealed that IL-33/ST2 axis may be related to both conditions; however, these associations were never evaluated in a single patients’ group. Here we explored the association among plasma levels of IL-33 and its decoy receptor soluble ST2 (sST2), carotid plaque determined by ultrasound, and volumetric bone mineral density (vBMD)/microstructure of distal radius measured by high-resolution peripheral quantitative computed tomography (HR-pQCT) in 80 PsA patients (55% male; 53.0 ± 10.1 years). Plasma sST2 levels were significantly higher in 33 (41%) patients with carotid plaques (11.2 ± 4.5 vs 7.7 ± 3.7 ng/ml, *P* < 0.001). In multivariate analysis, sST2 was an independent explanatory variable associated with carotid plaques (OR = 1.296, 95% CI: [1.091,1.540]; *P* = 0.003). After adjustment for the osteoporotic risk factors, sST2 was significantly associated with higher cortical porosity (β = 0.184, [0.042,0.325]; *P* = 0.012) and cortical pore volume (2.247, [0.434,4.060]; *P* = 0.016); and had a trend to be associated with lower cortical vBMD (−2.918, [−6.111,0.275]; *P* = 0.073). IL-33 was not associated with carotid plaque or vBMD/microstructure. In conclusion, plasma sST2 levels were independently correlated with both carotid plaque and compromised cortical vBMD/microstructure in PsA patients. IL-33/ST2 axis may be a link between accelerated atherosclerosis and osteoporosis in PsA.

Association between atherosclerosis and osteoporosis has been reported^1^,^2^,^3^. In a population-based study which included over 5,000 middle-to-advanced age subjects, low bone mineral density (BMD) of the forearm was associated with an increased risk of carotid atherosclerosis^1^. Similar findings were observed in patients with systemic lupus erythematosus and primary Sjogren’s syndrome^2^,^3^. Atherosclerosis and osteoporosis are common diseases accompanying the aging process, and share common risk factors, including metabolic disorders and chronic inflammation^4^. Whether there are dysregulated pathways which contribute to both conditions remain for further investigation.

Interleukin-33 (IL-33) is a recently discovered member of the Interleukin-1 (IL-1) cytokine family^5^. It could be released upon cell injury and served as an alarmin, or act extracellularly as a ligand for the IL-1 receptor family member ST2. The binding of IL-33 to ST2 further activates downstream pathways, leading to increased transcription of Th2 cytokines^5^. The ST2 receptors have two isoforms, a soluble (sST2) and a membrane bound form (ST2L). sST2 is an alternative spliced product of ST2, which lacks the transmembrane and intercellular domains. Thus, sST2 acts as a decoy receptor for IL-33 and inhibits IL-33/ST2 axis^5^. Emerging evidences suggest that this axis may play an essential role in both atherosclerosis and osteoporosis^6^,^7^. Nonetheless, whether this axis is a common pathway leading to both conditions in a same patient cohort has never been explored.

Psoriatic arthritis (PsA) is a chronic inflammatory arthritis associated with psoriasis^8^. Patients with PsA have an increased risk of subclinical atherosclerosis^9^,^10^. A recent meta-analysis revealed that PsA patients had an higher frequency of having carotid plaque compared with healthy controls [odds ratio (OR): 3.12]^10^. Although studies on the areal BMD (aBMD) by dual-energy X-ray absorptiometry (DXA) yielded conflicting results^11^,^12^,^13^, two recent studies evaluated the bone quality of distal radius using high-resolution peripheral quantitative computed tomography (HR-pQCT) and found compromised volumetric BMD (vBMD) and microstructure in PsA patients^14^,^15^. This may be due to the advantages that HR-pQCT assesses the 3 dimensional structures in a high spatial resolution of 130 μm, while DXA projects structures onto 2 dimensional images^16^. We hypothesize that the IL-33/ST2 axis may be one of the potential pathways providing the link between atherosclerosis and osteoporosis in PsA patients.

In this study, we aimed to elucidate the association among plasma IL-33 and sST2 levels and (i) the presence of carotid plaque using high-resolution ultrasound, and (ii) vBMD/bone microstructure of distal radius using HR-pQCT in PsA patients.

## Results

### Clinical features of PsA patients

A total of 80 (44 male, 55.0%) PsA patients were included. The mean age was 53.0 ± 10.1 years and disease duration was 14.3 ± 7.2 years. Table 1 summarized the anthropometric and clinical features of all the patients. IL-33 was detectable in 12 (15.0%) patients and mean sST2 was 9.1 ± 4.4 ng/ml. 9 PsA patients had 15 previous cardiovascular (CV) events, including 6 cerebrovascular accidents, 4 transient ischemic attacks, and 5 ischemic heart diseases. The clinical characteristics of PsA patients without CV events were summarized in Supplementary Table 1.

### IL-33/ST2 axis, carotid plaque, and Carotid intima media thickness (IMT)

Carotid plaque(s) were identified in 33 (41.3%) patients. The patients with carotid plaques were older (57.2 ± 9.3 vs 50.1 ± 9.7 years, *P* = 0.002), had longer PsA disease duration (16.9 ± 6.8 vs 12.5 ± 6.9 years, *P* = 0.006), and an increased prevalence of high cardiovascular diseases (CVD) risk (Framingham 10-year CVD risk >10%: 63.6% vs 34.0%, *P* = 0.013) (Table 1). There was a trend suggesting an increased prevalence of hyperlipidemia (36.4% vs 19.1%, *P* = 0.085) and more patients were put on statins (27.3% vs 10.6%, *P* = 0.046) in patients with plaques. CV events were more common in patients with plaques (21.2% vs 4.3%, *P* = 0.029).

**Figure 1:**
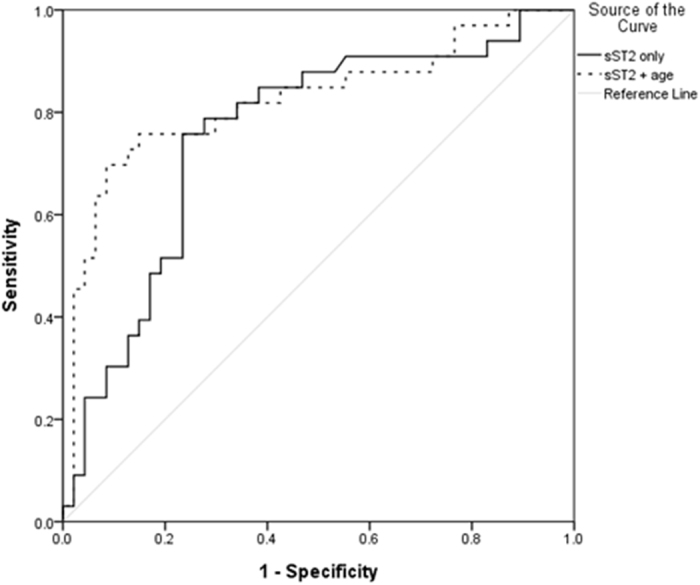
ROC curve for discriminating patients with or without carotid plaque (n = 80). The 2 independent risk factors in the multivariate analysis (sST2 and age) were used to generate a combined predictive score for plaque using logistic regression. Line: Plasma sST2 level only; AUROC: 0.757 (95% CI: [0.646, 0.867]; *P* < 0.001); Dots: Predictive value combined by plasma sST2 level and age; AUROC: 0.823 (95% CI: [0.723, 0.924]; *P* < 0.001).

Plasma sST2 levels were 11.2 ± 4.5 and 7.7 ± 3.7 ng/ml in patients with and without carotid plaques, respectively (*P* < 0.001). There was no difference in the prevalence of detectable IL-33 between patients with or without plaques (*P* = 0.752). After adjusting for the confounding factors, sST2 was an independent explanatory variable associated with the presence of carotid plaques (OR: 1.296, 95% confidence interval [CI]: [1.091, 1.540]; *P* = 0.003) (Table 2). Older age was another independent explanatory variable (1.094, [1.006, 1.189]; *P* = 0.036). sST2 levels discriminated PsA patients with or without plaques with area under receiver operating characteristic curve (AUROC) of 0.757 (95% CI: [0.646, 0.867]; *P* < 0.001) (Fig. 1). When age was added into the model, the combined predictive probability of sST2 and age increased the AUROC to 0.823 (0.723, 0.924). The results remained similar if patients with CV events were excluded (Supplementary Tables 1 and 2; Supplementary Figure 1).

On the other hand, mean or maximal IMT was not associated with plasma IL-33 or sST2 (all *P* > 0.1).

### IL-33/ST2 axis, vBMD and microstructure

Linear regression analysis showed that plasma sST2 was negatively associated with cortical vBMD (coefficient: −5.406, 95%CI [−8.550, −2.263]; *P* = 0.001), cortical tissue mineral density (TMD) (−2.538, [−4.540, −0.536]; *P* = 0.014), and positively associated with cortical porosity (Ct. Po) (0.248, [0.116, 0.380]; *P* < 0.001) and pore volume (2.973, [1.371, 4.575]; *P* < 0.001) (Table 3). The associations remained significant after adjusting for age and gender except for cortical TMD. After adjusting for the full model (age, gender, body mass index [BMI], hypertension, diabetes, erythrocyte sedimentation rate [ESR], c-reactive protein [CRP]), sST2 was still significantly associated with higher Ct. Po (0.184, [0.042, 0.325]; *P* = 0.012) and pore volume (2.247, [0.434, 4.060]; *P* = 0.016), and had a trend to be associated with lower cortical vBMD (−2.918, [−6.111, 0.275]; *P* = 0.073) (Table 3). Plasma sST2 levels were positively associated with vBMD for the peripheral region adjacent to the cortex (pTb) and trabecular number, and were negatively associated with trabecular separation in univariate analysis. However, only the positive association between sST2 and trabecular number remained significant after adjustment for age/gender, as well as for the full model (Table 3).

On the other hand, detectable IL-33 was not associated with the vBMD or microstructure at distal radius.

### Carotid plaque, vBMD and microstructure

In univariate analysis, PsA patients with carotid plaques had lower total vBMD (difference vs patients without plaque: −10.9%, *P* = 0.024), trabecular vBMD (−11.6%, *P* = 0.074), vBMD for the central medullary region (mTb) (−18.2%, *P* = 0.047), trabecular bone volume fraction (BV/TV) (−11.6%, *P* = 0.075) and trabecular thickness (−9.6%, *P* = 0.044). These differences remained significant after adjusting for age/gender or full adjustment except for total vBMD (all *P* < 0.05, Table 4).

## Discussion

This is the first study which evaluated the IL-33/ST2 axis, atherosclerosis and bone quality in a single patients’ group. We hypothesized that IL-33/ST2 axis could be a link between atherosclerosis and compromised bone quality in PsA patients. We found that plasma levels of sST2, the decoy receptor of IL-33, were independently associated with both the presence of carotid plaque and compromised cortical vBMD/microstructure, which supported the hypotheses. Our results further extended the previously isolated knowledge about the role of this axis on each condition^6^,^7^, by exploring the the overall association among IL-33/ST2 axis, atherosclerosis and osteoporosis.

Plasma IL-33 was detectable in 15% of the PsA patients. A previous study was unable to detect IL-33 in PsA patients, however, the case number was small (n = 7)^17^. It is possible that IL-33 may have formed immune complexes with sST2 or is downregulated by sST2 through negative regulatory mechanisms; however, this hypothesis remains unproven until a validated method for sST2-IL-33 complex assay is available^18^. Nevertheless, the low detection rate could mask the direct correlation between IL-33 and atherosclerosis or compromised bone quality, and limit the utility of blood IL-33 as a biomarker in PsA.

On the other hand, all patients had detectable plasma sST2. The association between plasma sST2 levels and carotid plaque was independent of traditional CV risk factors and the presence of CVD. The overall accuracy of sST2 in predicting carotid plaque was high (76%), and could be further increased to 82% when age was added to the predictive model. These results indicated that sST2 may be a good biomarker for atherosclerosis in PsA patients. sST2 had been proposed as a novel biomarker for CV events^6^. Although no association between sST2 and carotid atherosclerosis^19^,^20^,^21^ was found in the general population, it was never evaluated in PsA patients before. Moreover, we had previously reported that baseline detectable IL-33 independently predicted carotid plaque progression after following-up for 1 year in patients with early rheumatoid arthritis (ERA)^22^. Whether the IL-33/ST2 axis may play an important role in acclerating atherogenesis in patients with chronic inflammatory arthritis would need to be confirmed in future studies.

This is the first study to report the association between sST2, vBMD and bone microstructures. In multivariate analysis, only the positive association between sST2 and cortical porosity and cortical pore volume remained significant. The association between sST2 with compromised bone quality in terms of low cortical vBMD and TMD and was attenuated by adjusting for traditional osteoporotic risk factors, suggesting that the latter might partially mediate the effect of inflammation on osteoporosis. More importantly, compromised cortical bone quality was also the featured pattern of bone microstructural deficits in our PsA patients compared with controls^15^.

The plasma sST2 was also positively associated with trabecular number. However, this result should be interpreted carefully. Identifying the transitional zone between cortical and trabecular region remained difficult using HR-pQCT^16^. Endocortical resorption may induce small erosions or pores to the cortical bone in the endocortical region. These small cortical erosions/pores sometimes may be recognized incorrectly to be trabecular bone by HR-pQCT, which leads to increased trabecular number.

IL-33 and ST2 are expressed in the normal and atherosclerotic vasculature of murine and human^23^. Given atherosclerosis is a T helper (Th)1 immune response, IL-33/ST2 axis may have protective effects by inducing a Th1-to-Th2 switch of immune response. In Apolipoprotein E (ApoE) knock-out mice, IL-33 reduced the development of atherosclerosis via a Th1-to-Th2 switch^23^ and reducing accumulation of macrophage-derived foam cells in atherosclerotic plaques^24^. sST2 co-treatment was associated with the development of significantly larger atherosclerotic plaque and increased Th1 response. Increased sST2 was associated with worse cardiovascular outcomes in acute myocardial infarction^25^. In our ERA cohort, sST2 also tended to be elevated in patients with plaque progression, although it did not reach statistical significant^22^. However, there are also evidence suggesting that IL-33 was involved in the development of endothelial dysfunction and enhancement of adhesion molecular production^26^,^27^. Whether sST2 is associated with atherosclerotic plaque progression or CV events in PsA patients would need to be confirmed by future prospective studies.

IL-33 inhibited receptor activator of NF-kB ligand (RANKL)-induced osteoclast formation through the regulation of anti-osteoclastic genes such as interferon regulatory factor-8 (IRF-8), which could be blocked by an anti-ST2 monoclonal antibody^28^. Schulze *et al*. reported that IL-33 was expressed in osteoblasts, and prevented osteoclast formation from bone marrow precursor cells^29^. ST2 knock-out mice displayed increased bone resorption^29^, while IL-33 over-expression mice had significantly reduction in the number of osteoclasts^30^. Similar anti-osteogenic role of IL-33 was also reported by two other independent groups^31^,^32^. On the other hand, there were also few studies showing that IL-33 may promote osteoclastogenesis^33^,^34^. Nevertheless, the knowledge in the role of IL-33/ST2 axis in bone remolding is still limited. Our study may provide evidence to support the hypothesis that this axis may play an important role in bone loss in patients with PsA. Further studies are required to confirm this important question.

We also confirmed an association of atherosclerosis and compromised bone quality in PsA patients for the first time. However, the patients with carotid plaque had mainly compromised trabecular instead of cortical bone quality. It indicates that the extract underlying mechanism could be complicated and may not be explained by a single IL-33/ST2 axis. Other potential links between these two conditions, including chronic inflammation (sST2 was not correlated with inflammatory markers such as ESR [Spearman’s rho: −0.05, *P* = 0.665] or CRP [0.18, *P* = 0.115] in this cohort) and other important pathways, such as Wnt signaling pathway^35^, should be further evaluated.

The unique contribution of our study is the evaluation of both atherosclerosis and bone quality in one group of patients. Moreover, the bone quality was assessed by HR-pQCT, which allowed us to study the vBMD and microstructure of bone. Indeed, we could not find any correlation between aBMD and IL-33 or sST2 (data not shown). Our study also has a few limitations. The lack of control subjects does not allow us to test the associations in general population. Since this is an observational cross-sectional study, causal inferences cannot be drawn. Increased sST2 may either be the culprit or a compensatory response, or even an “innocent bystander” to the pathogenesis of atherosclerosis and osteoporosis. However, the clinical correlation highlighted the potential utility of sST2 as a biomarker, and further studies should be performed for a better understanding of the underlying mechanism. It is also important to note the presence of carotid plaque may incompletely capture the complex process of atherosclerosis in multiple vascular beds^36^, and interval carotid IMT and plaque measurements may confer more specificity in this process and relation with atherosclerotic outcomes. Last but not least, the associations should also be confirmed in other ethnical populations.

In conclusion, plasma sST2 levels were independently associated with both carotid plaque and compromised cortical vBMD/microstructure in PsA patients, indicating that IL-33/ST2 axis may contribute to both atherosclerosis and osteoporosis. These associations were independent of other established biomarkers including CRP and other established CV/osteoporotic risk factors. Future studies are warranted to elucidate the potential use of sST2 for screening and management of patients with subclinical atherosclerosis and possibly osteoporosis. The detailed mechanism should be further investigated.

## Patients and Methods

### Patients

Eighty consecutive PsA patients fulfilled the Classification of Psoriatic Arthritis criteria were recruited from the rheumatology clinic of The Prince of Wales Hospital for this cross-sectional study. The vBMD and microstructure of 53 out of the 80 patients comparing with age and gender matched controls had been published before^15^. Exclusion criteria were history of a disorder or using a drug that could affect bone metabolism; and pregnant or breastfeeding^15^. Treatment with glucocorticoids, calcium, and/or vitamin supplement was allowed. Patients with coexisting CV risk factors, and nine patients who had cardiovascular diseases CVD were also recruited. A subgroup analysis excluding these 9 patients was performed when evaluating the association between IL-33/ST2 axis and carotid plaque.

Ethics approval was obtained from Ethics Committee of The Chinese University of Hong Kong-New Territories East Cluster Hospitals (reference number: CRE-2012.082), and written informed consent was obtained from all participants according to the Declaration of Helsinki. The study was conducted in accordance with ICH-GCP.

### Clinical interview and laboratory tests

Clinical interview was performed by experienced rheumatologists using standardized data collection instruments as described before^37^. Smoking and drinking habits, disease history, family history and drug history were also retrieved. ESR, CRP, fasting blood glucose, and lipid profile were checked.

### Quantitative analysis of IL-33, sST2

Ten milliliters fasting ethylenediaminetetraacetic acid (EDTA) blood was collected. The EDTA blood samples were centrifuged at 2,000 *g* for 15 minutes to obtain plasma. Plasma samples were stored at −70 °C immediately until analysis. Plasma IL-33 and sST2 levels were measured using Human IL-33 Quantikine enzyme-linked immunosorbent assay (ELISA) Kit and Human ST2/IL-1 R4 Quantikine ELISA Kit (R&D Systems, Minneapolis, MN, USA) in singlicate. According to the manufacturer’s instruction, the minimum detectable dose of IL-33 and sST2 was 0.519 pg/mL and 5.1 pg/mL, respectively. The intra-assay coefficient of variation (CV) was about 3% for IL-33 and 5% for sST2. The inter-assay CV was about 5% for IL-33 and 6% for sST2, respectively.

### IMT and plaque

Carotid mean and maximum IMT were measured at 6 carotid arterial segments using a high-resolution B mode ultrasound machine (iE33; Philips, Andover, MA, USA) by an experienced cardiologist (QS) as described before^38^. Briefly, duplex carotid ultrasound was performed using an 11-MHz linear vascular probe. The IMT was measured offline in the distal common carotid artery, bulb, and proximal internal carotid artery using dedicated software (QLab 6.0; Philips). The mean and maximum of which were calculated. Plaque was defined as a localized thickening >1.2 mm. Our study involved a single ultrasonographer and a single reader. The intraclass correlation coefficient for the mean of the site-specific IMT values was 0.97^9^,^38^.

### HR-pQCT scanning and image analyses

vBMD and microstructure were measured at the distal radius of the non-dominant forearm using HR-pQCT (Scanco Medical AG, Bruettisellen, Switzerland) as described before^15^,^39^. The participant’s forearm was fixed in a carbon fiber cast, and a dorsal-palmer projection image was obtained to define the tomographic scan region. The scan region was default, i.e. 9.5 mm proximal from the mid-joint line and spanned 9.02 mm proximally in length, equivalent to a stack of 110 slices.

Images were first analyzed using a standard protocol provided by the manufacturer^15^. A semiautomated contouring process was used to segment the entire volume of interest into cortical and trabecular components, yielding average and trabecular vBMD in milligrams hydroxyapatite (HA) per cubic centimeter. pTb. vBMD, mTb. vBMD, BV/TV, trabecular number, thickness, separation and inhomogeneity were calculated accordingly. Our short-term HR-pQCT reproducibility, expressed as coefficient of variance, ranges from 0.38 to 1.03% for density measures and from 0.80 to 3.73% for microstructural measures^39^.

For indices of cortical volumetric density and microstructure, a fully automated cortical compartment segmentation technique was used^40^. Cortical vBMD and TMD were calculated. Indices of cortical microstructure included cortical thickness, cortical pore volume, Ct. Po, and pore diameter.

### Statistical analysis

Results were expressed as mean ± SD or median (interquartile range) as appropriate. Plasma IL-33 level was only expressed as detectable or un-detectable. Comparisons between 2 groups were assessed using the student’s t test or Mann-Whitney U test for continuous variables and χ^2^ test for categorical variables. Univariate and multivariable logistic regression analysis were performed to determine the independent predictor for the presence of carotid plaque. All variables with *P* < 0.1 in the univariate analysis were included in the multivariate analysis as potential confounding factors. Binary logistic regression was also used to calculate the predicted probability of combined utility of sST2 and age. ROC curve was used to calculate the accuracy in discriminating patients with or without carotid plaques. Univariate and multivariate linear regression analysis were used to assess the correlation between sST2 and HR-pQCT parameters. These associations were adjusted for age and gender, as well as clinical characteristics which were associated with lower cortical vBMD of the distal radius in PsA patients as we reported before (full adjustment model including: age, gender, BMI, hypertension, diabetes, ESR, CRP)^15^. Analysis of covariance (ANCOVA) was used to compare HR-pQCT parameters between patients with or without carotid plaque, adjusting for potential confounding factors. All statistical analyses were conducted using IBM SPSS Statistics Version 22 (IBM, Armonk, NY, USA). A minimal level of significance of *P* < 0.05 is used.

## Additional Information

**How to cite this article**: Shen, J. *et al*. Carotid plaque and bone density and microarchitecture in psoriatic arthritis: the correlation with soluble ST2. *Sci. Rep.* **6**, 32116; doi: 10.1038/srep32116 (2016).

## Supplementary Material

Supplementary Information

## Figures and Tables

**Table 1:** Anthropometric and clinical characteristics of patients.

	All (n = 80)	Carotid plaque		*P* value
		No (n = 47)	Yes (n = 33)	
Male gender, n (%)	44 (55.0%)	23 (48.9%)	21 (63.6%)	0.235
Age (years)	**53.0 ± 10.1**	**50.1 ± 9.7**	**57.2 ± 9.3**	**0.002**
PsA characteristics				
PsA disease duration (years)	**14.3 ± 7.2**	**12.5 ± 6.9**	**16.9 ± 6.8**	**0.006**
Tender joint count (0–68)	0 (0–3)	0 (0–3)	0 (0–3)	0.783
Swollen joint count (0–66)	0 (0–1)	0 (0–1)	0 (0–1)	0.359
Damaged joint count (0–68)	2 (0–6)	2 (0–6)	2 (1–8)	0.273
VAS Pain (0–100)	30 (10–58)	30 (20–50)	30 (10–60)	0.844
Patients’ global (0–100)	50 (20–60)	50 (20–60)	50 (20–60)	0.833
Physicians’ global (0–100)	20 (8–40)	20 (6–37)	20 (10–40)	0.746
PASI (0–72)	2.2 (0.8–6.4)	2.7 (0.9–6.5)	1.8 (0.5–5.4)	0.490
HAQ (0–3)	0.19 (0–0.63)	0.13 (0–0.50)	0.38 (0–0.88)	0.976
MDA, n(%)	16 (20.0%)	10 (21.3%)	6 (18.2%)	0.784
ESR (mm/1^st^ hr)	15 (7–28)	16 (7–31)	14 (6–28)	0.899
CRP (mg/dl)	0.3 (0.1–0.7)	0.3 (0.1–0.6)	0.3 (0.1–0.8)	0.799
CV and osteoporosis risk factors				
Body weight (kg)	68.5 ± 13.6	68.5 ± 15.3	68.7 ± 10.9	0.944
BMI (kg/m^2^)	25.6 ± 4.1	25.8 ± 4.5	25.4 ± 3.5	0.675
Systolic BP (mmHg)	129 ± 17	128 ± 15	130 ± 19	0.614
Diastolic BP (mmHg)	82 ± 11	82 ± 10	81 ± 12	0.593
Hypertension, n(%)	46 (57.5%)	26 (55.3%)	20 (60.6%)	0.721
Diabetes, n(%)	16 (20.0%)	7 (14.9%)	9 (27.3%)	0.151
Hyperlipidemia, n(%)	21 (26.3%)	9 (19.1%)	12 (36.4%)	0.085
Metabolic syndrome, n(%)	23 (29.1%)	15 (32.6%)	8 (24.2%)	0.469
Framingham 10-year CVD risk > 10%, n(%)	**37 (46.3%)**	**16 (34.0%)**	**21 (63.6%)**	**0.013**
Cardiovascular event, n(%)	**9 (11.3%)**	**2 (4.3%)**	**7 (21.2%)**	**0.029**
Total cholesterol (mmol/L)	5.0 ± 0.8	5.0 ± 0.8	5.0 ± 0.8	0.878
HDL cholesterol (mmol/L)	1.4 ± 0.4	1.4 ± 0.4	1.4 ± 0.4	0.841
Triglycerides (mmol/L)	1.5 ± 0.9	1.6 ± 1.0	1.4 ± 0.6	0.572
Fasting glucose (mmol/L)	5.6 ± 1.7	5.7 ± 1.9	5.5 ± 1.4	0.542
Post-menopause women, n(%)	22 (61.1%)	14 (58.3%)	8 (66.7%)	0.727
Current smoker, n(%)	9 (11.3%)	5 (10.6%)	4 (12.1%)	0.999
Current drinker, n(%)	1 (1.3%)	1 (2.1%)	0 (0%)	0.999
Parent history of fracture, n(%)	7 (8.8%)	5 (10.6%)	2 (6.1%)	0.694
History of fracture, n(%)	7 (8.8%)	4 (8.5%)	3 (9.1%)	0.999
Current medications, n (%)				
Anti-hypertensive	44 (55.0%)	25 (53.2%)	19 (57.6%)	0.698
Statins	**14 (17.5%)**	**5 (10.6%)**	**9 (27.3%)**	**0.046**
NSAIDs	34 (42.5%)	21 (44.7%)	13 (39.4%)	0.525
Steroids	2 (2.5%)	1 (2.1%)	1 (3.0%)	0.999
DMARDs	45 (56.3%)	28 (59.6%)	17 (51.5%)	0.400
Biologics	14 (17.5%)	8 (17.0%)	6 (18.2%)	0.843
Biomarkers				
Detectable IL-33, n (%)	12 (15.0%)	8 (17.0%)	4 (12.1%)	0.752
sST2 (ng/ml)	**9.1 ± 4.4**	**7.7 ± 3.7**	**11.2 ± 4.5**	** < 0.001**

VAS: visual analogue scale, PASI: Psoriasis Area and Severity Index, HAQ: Health Assessment Questionnaire, MDA: minimal disease activity, ESR: erythrocyte sedimentation rate, CRP: c-reactive protein, CV: cardiovascular, BMI: body mass index, BP: blood pressure, HDL: high-density lipoprotein, NSAIDs: nonsteroidal anti-inflammatory drugs, DMARDs: disease-modifying antirheumatic drugs. Values are the number (percentage) or median (interquatile range) or mean ± SD.

**Table 2:** Univariate and multivariate analysis for factors associated with presence of carotid plaque.

	Univariate			Multivariate*		
	OR	95% CI	*P* value	OR	95% CI	*P* value
Age (years)	1.087	1.027–1.087	0.004	**1.094**	**1.006**–**1.189**	**0.036**
Disease duration (years)	1.094	1.023–1.171	0.009	1.065	0.983–1.154	0.125
Hyperlipidemia, n(%)	2.413	0.874–6.660	0.089	2.147	0.368–12.541	0.396
Framingham 10-year CVD risk >10%, n(%)	3.391	1.336–8.604	0.010	0.958	0.269–3.414	0.947
Current use of statins, n(%)	3.150	0.946–10.487	0.062	0.851	0.092–7.916	0.888
Cardiovascular event, n(%)	6.058	1.170–31.353	0.032	2.060	0.265–16.038	0.490
sST2 (ng/ml)	1.246	1.091–1.422	0.001	**1.296**	**1.091**–**1.540**	**0.003**

^*^Factors included in the multivariate analysis: age, disease duration, hyperlipidemia, framingham 10-year CVD risk >10%, current use of statins, cardiovascular event, and sST2.

**Table 3:** Univariate and multivariate linear regression analysis for the associations among plasma sST2 and HR-pQCT parameters.

	Unadjusted		Adj. for age, gender		Adj. for age, gender, BMI, HT, DM, ESR, CRP	
	Coefficients (95%CI)	*P* value	Coefficients (95%CI)	*P* value	Coefficients (95%CI)	*P* value
vBMD, mgHA/cm^3^						
Average vBMD	−0.129 (−4.236, −3.978)	0.950				
Ct. vBMD	**−5.406 (−8.550, −2.263)**	**0.001**	**−4.603 (−7.835, −1.372)**	**0.006**	−2.918 (−6.111, 0.275)	0.073
Tb. vBMD	2.107 (−0.151, 4.366)	0.067	0.895 (−1.425, 3.214)	0.445		
pTb. vBMD	**2.535 (0.355, 4.715)**	**0.023**	1.518 (−0.751, 3.786)	0.187		
mTb.vBMD	1.833 (−0.541, 4.206)	0.128				
Trabecular microstructure						
BV/TV	0.002 (0.000, 0.004)	0.068	0.001 (−0.001, 0.003)	0.450		
Tb. number, mm^−1^	**0.026 (0.011, 0.042)**	**0.001**	**0.023 (0.006, 0.040)**	**0.009**	**0.024 (0.006, 0.041)**	**0.008**
Tb. thickness, mm	0.000 (−0.001, 0.001)	0.897				
Tb. separation, mm	**−0.010 (−0.019, −0.001)**	**0.034**	−0.006 (−0.017, 0.004)	0.208		
Inhomogeneity, mm	−0.004 (−0.011, 0.003)	0.238				
Cortical microstructure						
Ct. TMD, mgHA/cm^3^	**−2.538 (−4.540, −0.536)**	**0.014**	−1.791 (−3.894, 0.311)	0.094	−0.723 (−2.808, 1.363)	0.492
Ct. thickness, mm	0.009 (−0.007, 0.024)	0.257				
Ct. PoV, mm^3^	**2.973 (1.371, 4.575)**	**<0.001**	**2.917 (1.142, 4.693)**	**0.002**	**2.247 (0.434, 4.060)**	**0.016**
Ct. Po, %	**0.248 (0.116, 0.380)**	**<0.001**	**0.246 (0.105, 0.386)**	**0.001**	**0.184 (0.042, 0.325)**	**0.012**
Ct. Po. Dm, mm	−0.001 (−0.002, 0.001)	0.410				

vBMD: volumetric bone mineral density, Ct.: cortical, Tb.: trabecular, TMD: tissue mineral density, Po: porosity index, PoV: pore volume, Po. Dm: pore diameter, BMI: body-mass index, HT: hypertension, DM: diabetes, ESR: erythrocyte sedimentation rate, CRP: c-reactive protein.

**Table 4:** Carotid plaque and HR-pQCT parameters.

	Carotid plaque		% difference	*p* value	*p* value adjusted for	
	No (n = 47)	Yes (n = 33)			Age, gender	Age, gender, BMI, HT, DM, ESR, CRP
vBMD, mgHA/cm^3^						
Average vBMD	**369.6 ± 81.6**	**329.3 ± 70.5**	**−10.9**	**0.024**	0.080	0.121
Ct. vBMD	976.2 ± 67.1	951.1 ± 60.0	−2.6	0.090	0.762	
Tb. vBMD	**155.3 ± 48.3**	**137.2 ± 36.7**	**−11.6**	**0.074**	**0.026**	**0.038**
pTb. vBMD	213.1 ± 47.6	199.2 ± 35.9	−6.5	0.161		
mTb.vBMD	**115.0 ± 49.7**	**94.1 ± 39.0**	**−18.2**	**0.047**	**0.014**	**0.020**
Trabecular microstructure						
BV/TV	**0.129 ± 0.040**	**0.114 ± 0.031**	**−11.6**	**0.075**	**0.027**	**0.039**
Tb. number, mm^−1^	1.61 ± 0.034	1.58 ± 0.29	−2.2	0.630		
Tb. thickness, mm	**0.080 ± 0.018**	**0.072 ± 0.014**	**−9.6**	**0.044**	**0.031**	**0.029**
Tb. separation, mm	0.570 ± 0.155	0.597 ± 0.222	4.6	0.533		
Inhomogeneity, mm	0.253 ± 0.111	0.276 ± 0.171	9.3	0.457		
Cortical microstructure						
Ct. TMD, mgHA/cm^3^	1015 ± 40	999 ± 39	−1.5	0.093	0.662	
Ct. thickness, mm	1.17 ± 0.33	1.06 ± 0.23	−9.4	0.105		
Ct. PoV, mm^3^	21.6 ± 42.3	20.0 ± 14.1	−7.3	0.836		
Ct. Po, %	2.92 ± 3.14	3.43 ± 2.15	17.7	0.416		
Ct. Po. Dm, mm	0.19 ± 0.03	0.19 ± 0.02	0.2	0.941		

vBMD: volumetric bone mineral density, Ct.: cortical, Tb.: trabecular, TMD: tissue mineral density, Po: porosity index, PoV: pore volume, Po. Dm: pore diameter, BMI: body-mass index, HT: hypertension, DM: diabetes, ESR: erythrocyte sedimentation rate, CRP: c-reactive protein.
