# Supplementary material for: Carotid plaque and bone density and microarchitecture in psoriatic arthritis: the correlation with soluble ST2
Source: Sci Rep. 2016 Aug 24;6:32116. doi: 10.1038/srep32116 (PMC4995470; doi:10.1038/srep32116)
Supplement: Supplementary Information [file srep32116-s1.pdf]

Title: Carotid plaque and bone density and microarchitecture in psoriatic arthritis: the correlation with soluble ST2

Authors:

Jiayun Shen[1], PhD, Qing Shang[1], PhD, Chun-Kwok Wong[2], PhD, Edmund K. Li[1], MD, Emily W Kun[3], FRCP, Isaac T. Cheng[1], BSc, Martin Li[1], PhD, Tena K. Li[1], BN, Tracy Y. Zhu[4], PhD, Cheuk-Man Yu[1], MD, Ling Qin[4],PhD, Lai-Shan Tam*[1], MD.

Affiliated institution:

[1]Department of Medicine & Therapeutics, The Prince of Wales Hospital, The Chinese University of Hong Kong, Hong Kong; [2]Department of Chemical Pathology, The Prince of Wales Hospital, The Chinese University of Hong Kong, Hong Kong, [3]Department of Medicine and Geriatrics, Taipo Hospital, Hong Kong, [4]Bone Quality and Health Center of the Department of Orthopedics & Traumatology, The Prince of Wales Hospital, The Chinese University of Hong Kong, Hong Kong.

Supplementary Table 1. Anthropometric and clinical characteristics of patients without cardiovascular diseases

	All (n=71)	Carotid plaque		*P* value
		No (n=45)	Yes (n=26)	
Male gender, n(%)	39(54.9%)	22(48.9%)	17(65.4%)	0.178
Age (years)	**51.7±9.5**	**49.7±9.8**	**55.2±7.9**	**0.018**
PsA characteristics				
PsA disease duration (years)	**13.9±7.0**	**12.1±6.8**	**17.0±6.4**	**0.004**
Tender joint count (0-68)	0(0-2)	0(0-2)	0(0-1)	0.460
Swollen joint count (0-66)	0(0-1)	0(0-0)	0(0-2)	0.149
Damaged joint count (0-68)	2(0-6)	2(0-4)	2(0-9)	0.176
VAS Pain (0-100)	30(10-50)	30(20-50)	30(10-60)	0.947
Patients’ global (0-100)	50(20-60)	40(20-60)	50(30-60)	0.368
Physicians’ global (0-100)	20(7-40)	20(6-35)	23(10-40)	0.461
PASI (0-72)	2.2 (0.7-6.5)	2.7 (0.9-6.2)	1.8 (0.5-10.8)	0.862
HAQ (0-3)	0.13(0-0.50)	0.13(0-0.38)	0.31(0-0.75)	0.225

MDA, n(%)	13(18.3%)	10(22.2%)	3(11.5%)	0.348
ESR (mm/1st hr)	15(7-28)	15(7-29)	15(6-28)	0.831
CRP (mg/dl)	0.3(0.1-0.7)	0.3(0.1-0.6)	0.4(0.1-0.8)	0.391
CV and osteoporosis risk factors				
Body weight (kg)	68.2±14.0	68.6±15.5	67.4±11.0	0.725
BMI (kg/m^2)	25.5±4.3	25.8±4.6	24.9±3.6	0.385
Systolic BP (mmHg)	128±15	127±15	128±16	0.779
Diastolic BP (mmHg)	82±10	82±10	82±11	0.917
Hypertension, n(%)	38(53.5%)	24(53.3%)	14(53.8%)	0.967
Diabetes, n(%)	11(15.5%)	6(13.3%)	5(19.2%)	0.516
Hyperlipidemia, n(%)	17(23.9%)	8(17.8%)	9(34.6%)	0.109
Metabolic syndrome, n(%)	20(28.2%)	14(31.1%)	6(23.1%)	0.434
Framingham 10-year CVD risk > 10%, n(%)	**31(43.7%)**	**15(33.3%)**	**16(61.5%)**	**0.021**
Total cholesterol (mmol/L)	5.0±0.9	5.0±0.8	5.1±0.9	0.601
HDL cholesterol (mmol/L)	1.4±0.4	1.4±0.4	1.4±0.4	0.863
Triglycerides (mmol/L)	1.5±0.9	1.5±1.1	1.4±0.6	0.368
Fasting glucose (mmol/L)	5.6±1.8	5.7±1.9	5.4±1.6	0.463

Post-menopause women, n(%)	18(56.3%)	13(56.5%)	5(55.6%)	0.999
Current smoker, n(%)	8(11.3%)	5(11.1%)	3(11.5%)	0.999
Current drinker, n(%)	1(1.4%)	1(2.2%)	0(0%)	0.999
Parent history of fracture, n(%)	7(9.9%)	5(11.1%)	2(7.7%)	0.999
History of fracture, n(%)	6(8.5%)	3(6.7%)	3(11.5%)	0.662
Current medications, n (%)				
Anti-hypertensive	36(50.7%)	23(51.1%)	13(50.0%)	0.928
Statins	10(14.1%)	4(8.9%)	6(23.1%)	0.155
NSAIDs	32(45.1%)	21(46.7%)	11(42.3%)	0.722
Steroids	2(2.8%)	1(2.2%)	1(3.8%)	0.999
DMARDs	40(56.3%)	27(60.0%)	13(50.0%)	0.413
Biologics	11(15.5%)	7(15.6%)	4(15.4%)	0.999
Biomarkers				
Detectable IL-33, n (%)	11(15.5%)	8(17.8%)	3(11.5%)	0.735
sST2 (ng/ml)	**8.7±3.8**	**7.4±3.0**	**10.9±3.9**	**<0.001**

VAS: visual analogue scale, PASI: Psoriasis Area and Severity Index, HAQ: Health Assessment Questionnaire, MDA: minimal disease activity, ESR:

erythrocyte sedimentation rate, CRP: c-reactive protein, CV: cardiovascular, BMI: body mass index, BP: blood pressure, HDL: high-density lipoprotein, NSAIDs: nonsteroidal anti-inflammatory drugs, DMARDs: disease-modifying antirheumatic drugs.

Values are the number (percentage) or median (interquatile range) or mean±SD.

Supplementary Table 2. Univariate and multivariate analysis for factors associated with presence of carotid plaque in patients without cardiovascular diseases (n=71)

	Univariate			Multivariate		
	OR	95% CI	*P* value	OR	95% CI	*P* value
Age (years)	1.072	1.009-1.138	0.024	**1.091**	**1.000-1.191**	**0.049**
Disease duration (years)	1.112	1.030-1.201	0.007	**1.100**	**1.004-1.205**	**0.041**
Framingham 10-year CVD risk > 10%, n(%)	3.200	1.172-8.735	0.023	0.991	0.239-4.112	0.991
sST2 (ng/ml)	1.334	1.129-1.576	0.001	**1.431**	**1.162-1.763**	**0.001**

Supplementary Figure 1. ROC curve for discriminating patients with or without carotid plaque in patients without cardiovascular diseases (n=71)

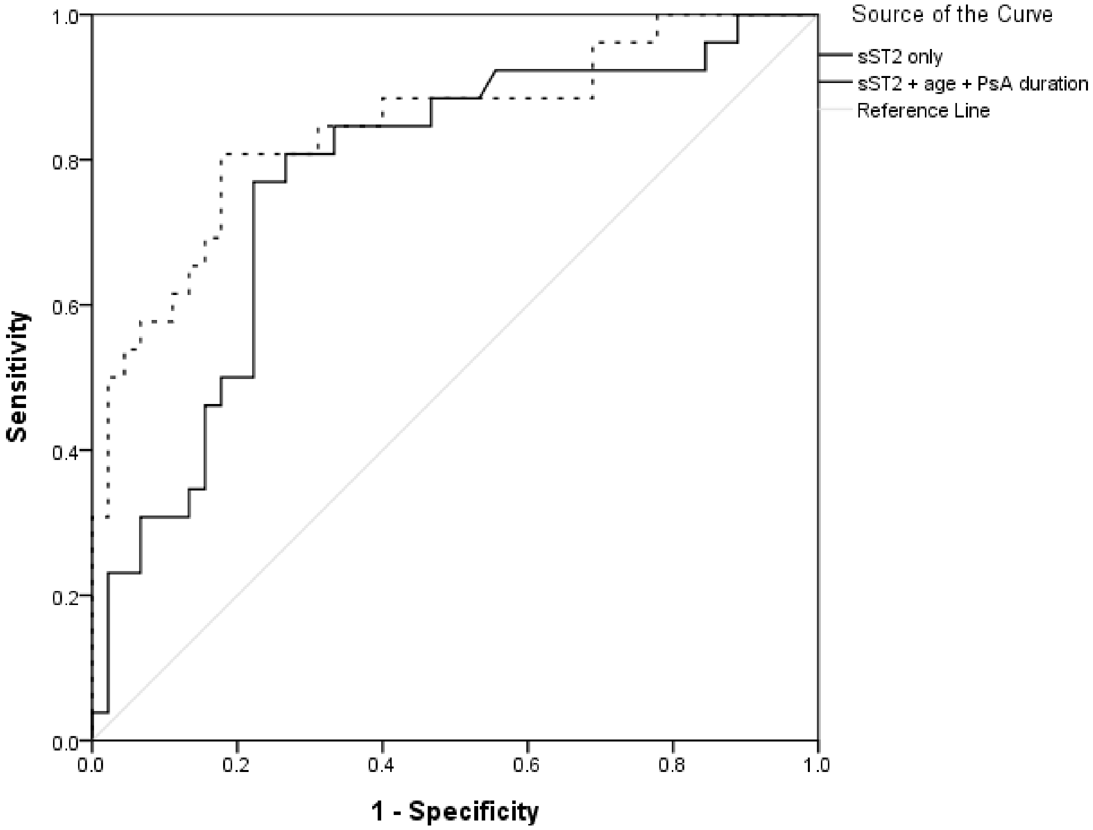

The 2 independent risk factors in the multivariate analysis (sST2 and age) were used to generate a combined predictive score for plaque using logistic regression. Line: Plasma sST2 level only; AUROC: 0.772 (95% CI: [0.657, 0.887]; *P* <0.001);

Dots: Predictive value combined by plasma sST2 level and age; AUROC: 0.845 (95% CI: [0.746, 0.945]; *P* <0.001).
